# Small proteins: untapped area of potential biological importance

**DOI:** 10.3389/fgene.2013.00286

**Published:** 2013-12-16

**Authors:** Mingming Su, Yunchao Ling, Jun Yu, Jiayan Wu, Jingfa Xiao

**Affiliations:** ^1^CAS Key Laboratory of Genome Sciences and Information, Beijing Institute of Genomics, Chinese Academy of SciencesBeijing, China; ^2^Graduate University of Chinese Academy of SciencesBeijing, China

**Keywords:** small proteins, small ORFs, protein identification, protein annotation coherence, evolution characterization

## Abstract

Polypeptides containing ≤100 amino acid residues (AAs) are generally considered to be small proteins (SPs). Many studies have shown that some SPs are involved in important biological processes, including cell signaling, metabolism, and growth. SP generally has a simple domain and has an advantage to be used as model system to overcome folding speed limits in protein folding simulation and drug design. But SPs were once thought to be trivial molecules in biological processes compared to large proteins. Because of the constraints of experimental methods and bioinformatics analysis, many genome projects have used a length threshold of 100 amino acid residues to minimize erroneous predictions and SPs are relatively under-represented in earlier studies. The general protein discovery methods have potential problems to predict and validate SPs, and very few effective tools and algorithms were developed specially for SPs identification. In this review, we mainly consider the diverse strategies applied to SPs prediction and discuss the challenge for differentiate SP coding genes from artifacts. We also summarize current large-scale discovery of SPs in species at the genome level. In addition, we present an overview of SPs with regard to biological significance, structural application, and evolution characterization in an effort to gain insight into the significance of SPs.

## Introduction

Proteins generally contain from 50 to 1000 amino acid residues (AAs) per polypeptide chain. In most studies, polypeptides containing ≤100 AAs are considered to be small proteins (SPs) but there is no strict definition of an SP. Some studies have used wider thresholds for SPs of ≤200 AAs (Yang et al., 2011) and some have used narrow thresholds for SPs of ≤85 AAs (Zuber, 2001; Schmidt and Davies, 2007). To date, the smallest protein described is the TAL protein (11 AAs), which influences development of the *Drosophila melanogaster* (Galindo et al., 2007). Because of the short length, SPs generally consist of a simple domain and represent simple, useful model systems for simulation of protein folding (Imperiali and Ottesen, 1999; Polticelli et al., 2001) and for drug design (Martin and Vita, 2000). But many of the earlier studies assumed that the length of a protein sequence is associated with its specific functions and that SPs probably have few notable functions compared to large proteins. According to a statistical survey of SPs, the majority of SPs in a certain species are hypothetical proteins or proteins with unknown functions (Wang et al., 2008), and it is less likely to find shorter proteins with confirmatory homology in other organisms (Lipman et al., 2002; Wang et al., 2008; Zhao et al., 2012). Large proteins have the priority to be annotated (Galperin and Koonin, 2004) and studied while shorter proteins to be relatively unimportant (Hirsh and Fraser, 2001; Jordan et al., 2002). However, the identification of increasing numbers of important SPs has gradually attracted the attention of scientists and many studies have demonstrated that SPs are widespread and have important functionality in all three domains of life (Camby et al., 2006; Galindo et al., 2007; Gleason et al., 2008; Muller et al., 2008; Notaguchi et al., 2008; Oelkers et al., 2008; Jung et al., 2009). In fact, due to binding studies of peptides of various sizes, the minimal size of a functional epitope is ~8AAs, with an average size of 15–20 AAs. SPs with less than 100 AA are sufficient to contain at least a single domain that exhibits a relevant function or to assist a biological process (Wang et al., 2008). Furthermore, there appears to be a significant evolutionary trend favoring shorter rather than longer proteins for specialized functions (Lipman et al., 2002). This field is receiving increasing interest focused on the significance of SPs. Thus, the bottleneck for the research on SPs might not be the “trivial” functional SPs themselves but the techniques of discovery and analysis of SPs.

## Small protein-coding genes overlooked in genome annotation

In pace with the rising sequence data in NCBI database, the biggest challenge for whole genome annotation and analysis is becoming to differentiate meaningful gene-coding ORFs from inutile ORFs. Random sequence simulation suggests that, except for long repetitive sequences, ORFs ≥200 AAs are unlikely to occur by chance, whereas a large number of sORFs could include numerous artificial genes (Fickett, 1995; Das et al., 1997). SP-coding genes could easily escape detection in a genome-wide prediction because they are “buried” in an enormous pile of sORFs (Basrai et al., 1997). Dujon et al. defined a key criterion to annotate an ORF; this criterion takes proteins with ≥100 contiguous codons (including the first ATG) as functional genes and ORFs that are shorter than 100 codons as questionable genes (Dujon et al., 1994). With the application of this criterion, ORFs were identified automatically in the yeast *Saccharomyces cerevisiae* chromosome XI (Dujon et al., 1994). Goffeau also applied this criterion and defined 5885 potential protein-encoding genes from the 12,068 Mb DNA sequence of the *S. cerevisiae* genome, exclusive of SPs (Goffeau et al., 1996). Since then, most algorithms of genome annotation or protein prediction have used a cutoff of ≤100 AAs to reduce the likelihood of false-positive genes. In 2006, Kastenmayer et al. used gene expression-based analyses and homology searching and brought 299 un-annotated sORFs in *S. cerevisiae*, 247 of which have been verified experimentally (Kastenmayer et al., 2006). Thus, the limitations of discovery techniques could have contributed to the assumption that the functions of SPs are less worthy of study. It is suggested that the number of SP-coding genes is substantially greater than those discovered to date, which becomes a challenging problem for biologists trying to predict and validate SPs throughout the genome.

## Functional significance of SPs

As a result of the constraints of discovery techniques, few SPs are identified and the majority of SPs are annotated as hypothetical proteins or proteins with unknown functions. But SPs with known functions are involved in various important functional classes, including information storage and processing, cellular processes and signaling, and metabolism. There is growing evidence that many sORFs in single-celled microorganisms surprisingly encode small bioactive peptides. Some of the well-known SPs include chaperonin, Hsp10, translation initiation factor IF-1, ribosomal proteins and others (Wang et al., 2008). In Bacteria, two SPs, PtrA and PtrB are actively participate in the suppression of the type III secretion system under the stress of DNA damage in *Pseudomonas aeruginosa* (Ha et al., 2004; Wu and Jin, 2005); a group of small acid-soluble spore proteins (SASP) are the crucial factors that protect spore DNA from damaging in dormant spores of *Bacillus*, *Clostridium*, and related species (Setlow, 2007). In yeast, it has been reported that SPs include mating pheromones, proteins involved in energy metabolism, proteolipids, chaperonins, stress proteins, transporters, transcriptional regulators, nucleases, ribosomal proteins, thioredoxins, and metal ion chelators (Basrai et al., 1997). In fact, regulatory and metabolic proteins are more common than constitutive or structural proteins. On the basis of the Clusters of Orthologous Groups (COG) database (Tatusov et al., 2000), we summarized the function types of SPs in Archaea, Bacteria, and Fungi and found that SPs cover nearly all subclasses of functional classes in the COG database, except for constitutive or structural classes; i.e., RNA processing and modification, nuclear structure, and extracellular structures (Table 1).

**Table 1:** **Function characterization of small proteins in COG/KOG database**.

**COG/KOG function classes**	***Archaea***	***Bacteria***	***Fungi***	***Cel***	***Ath***	***Dme***	***Hsa***
[J] Translation, ribosomal structure, and biogenesis	+	+	+	+	+	+	+
[A] RNA processing and modification	−	−	−	+	+	+	+
[K] Transcription	+	+	+	+	+	+	+
[L] Replication, recombination, and repair	+	+	−	+	+	+	+
[B] Chromatin structure and dynamics	+	+	+	+	+	+	+
[D] Cell cycle control, cell division, chromosome partitioning	+	+	+	+	+	+	+
[Y] Nuclear structure	−	−	−	−	−	−	−
[V] Defense mechanisms	+	+	−	+	−	+	+
[T] Signal transduction mechanisms	+	+	+	+	+	+	+
[M] Cell wall/membrane/envelope biogenesis	+	+	−	−	+	+	+
[N] Cell motility	+	+	−	+	−	+	+
[Z] Cytoskeleton	−	+	+	+	+	+	+
[W] Extracellular structures	−	−	−	+	+	+	+
[U] Intracellular trafficking, secretion, and vesicular transport	+	+	+	+	+	+	+
[O] Posttranslational modification, protein turnover, chaperones	+	+	+	+	+	+	+
[C] Energy production and conversion	+	+	+	+	+	+	+
[G] Carbohydrate transport and metabolism	+	+	+	+	+	−	+
[E] Amino acid transport and metabolism	+	+	−	+	+	+	+
[F] Nucleotide transport and metabolism	+	+	−	−	+	−	+
[H] Coenzyme transport and metabolism	+	+	+	+	+	−	+
[I] Lipid transport and metabolism	+	+	+	+	+	+	+
[P] Inorganic ion transport and metabolism	+	+	+	+	+	+	+
[Q] Secondary metabolites biosynthesis, transport, and catabolism	+	+	−	−	+	+	+
[R] General function prediction only	+	+	+	+	+	+	+
[S] Function unknown	+	+	+	+	+	+	+

“+,” found; “−,” not found. It describes function types of SPs in Archaea, Bacteria, and Fungi in COG database and those of SPs in eukaryotic species in KOG database. In Archaea, bacteria, and fungi, constitutive or structural classes are not covered, that is, RNA processing and modification, nuclear structure, extracellular structures. In Arabidopsis thaliana (Ath), Caenorhabditis elegans (Cel), Drosophila melanogaster (Dme), and Homo sapiens (Hsa), the nuclear structure class is not covered in all these organisms.

Among multicellular organisms, certain important signaling molecules, hormones, antibacterial defensins, animal toxins, and protease inhibitors belong to the SP family. In plants, some SPs are known to be involved in cell-to-cell communications and regulatory processes. It was demonstrated recently that a membrane-associated thioredoxin (140 AAs) (Meng et al., 2010) is related to intercellular communication, the Cg-1 protein (<33 AAs) (Gleason et al., 2008) controlling the tomato/nematode interaction, the lipid-binding protein AZI1(161 AAs) (Jung et al., 2009) involved in priming plant defenses, and the FLOWERING LOCUS T (FT) protein (175 AAs) (Notaguchi et al., 2008) acting as a long-range signal regulating flowering. In *Arabidopsis*, the CLE family proteins (75–140 AAs) (Fletcher et al., 1999; Trotochaud et al., 2000; Muller et al., 2008) participate in meristem development. CAPRICE (CPC; 94 AAs) is a transcription factor involved in intercellular signal transduction associated with root epidermal cell differentiation (Kurata et al., 2005). In animals, there is a rich diversity of short peptides involved in intercellular transportation and development (Basrai et al., 1997). A eukaryotic TAL protein (11 AA) was reported to influence Drosophila development (Galindo et al., 2007). A long non-coding RNA called polished rice (pri) was found to encode small peptides (11–32 AA) that control proteolytic cleavage of a transcription factor control Shavenbaby (Svb) during *Drosophila* embryogenesis (Kondo et al., 2010). In humans, galectin-1 (135 AAs), for example, plays major roles in neuronal cell differentiation and the establishment and maintenance of T-cell tolerance and homeostasis *in vivo* (Luo et al., 1996). In fact, it is clear that almost all subclasses of functional classes in KOG database (eukaryotic representatives of the COG database) (Tatusov et al., 2003) are covered in *A. thaliana* (*Ath*), *Caenorhabditis elegans* (*Cel*), *Drosophila melanogaster* (*Dme*), *and Homo sapiens* (*Hsa*), except for the nuclear structure class (Table 1; see Table S1 for details).

**Figure 1:**
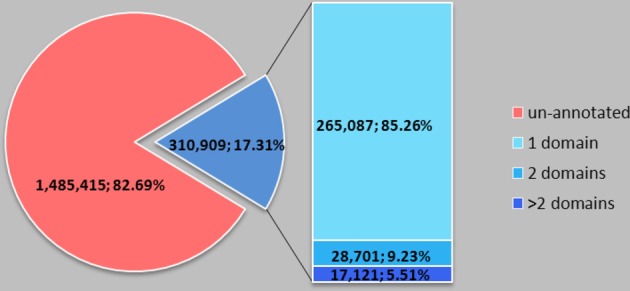
**Domain number distribution of small proteins in NCBI genpept**. SP usually contains a single domain. The NCBI genpept database contains 14,324,397 proteins, including 1,796,324 (12.54%) SPs. Only 310,909 (17.31%) SPs, about 2.17% of total proteins, are annotated, and among the annotated domain SPs, most of them (85.26%) have only one domain.

We further studied domains of SPs in the Pfam-A database (Pfam-A) and the NCBI genpept database (NCBI genpept database). The NCBI genpept database contains 14,324,397 proteins, including 1,796,324 (12.54%) SPs. Only 310,909 (17.31%) SPs, about 2.17% of total proteins are annotated, and among the annotated domain SPs, most of them (85.26%) have only one domain (Figure 1). SPs usually contain single domain. Domain-known SPs cover 3274 domain items (85.39% of the total 3834 domain items in all Pfam-A families against the NCBI genpept database) (Table S2). Some similar domains are grouped together into clans; we clustered the 3274 domains on the basis of Pfam-C, but not every domain has a corresponding clan. Specifically, 1687 domains belong to clans and 1587 domains were not found in clans (Table S3). Domain analysis revealed that large numbers of SPs are not identified but, as for SPs with known domain, they usually have a simple structure and cover almost all domain classes.

## Prediction and validation for SPs

Although recent advances in computational and experimental approaches make it possible to identify ORFs efficiently at the genome-wide level, there are potential problems for the prediction and validation for SPs.

### Homology-based searching

The general technique of homology-based gene prediction is the most reliable tool for discovering evolutionarily conserved genes. The key issue is to identify sequence similarity. Each predicted gene from the stringent genome sequence can be annotated if the gene aligns significantly with a known protein sequence from the same organism or other organisms. It has been used to discover small, un-annotated, protein-coding, and non-protein-coding genes in chromosomal regions previously considered to be intergenic region between the genome of *S. cerevisiae* and those of other hemiascomycetous yeasts (Blandin et al., 2000) or other Saccharomyces genomes (Cliften et al., 2001). However, as for SPs, the sequence similarity-based homology assessment method is limited by the large size of the protein database and the short length of the sequence. The expectation (*e*-value) for finding a random sequence match in a database often takes into account the length of the sequence and, for a short query sequence, the probability of a random match is quite high. Thus, alignment-based methods exclude a lot of small potential genes, which are often classified as ORFs that occur by chance (Skovgaard et al., 2001). Moreover, it is also less likely to find SPs that do not have confirmatory homology in other organisms. By BLAST comparisons, Wang F et al. took several representative phyla to investigate conservation among SPs and found the species-specific SPs are the majority in all of the phyla (Wang et al., 2008). Thus, homology searching is not competent for discovery of species-specific SPs. And SPs like ubiquitin (76 AA, not including the pre-protein peptide), which is highly conserved from fungi to mammals but only sharing a similar structure with ubiquitin proteins in prokaryotic cells, are also excluded by alignment-based approach (Bienkowska et al., 2003).

### Purely statistical algorithms

Purely statistical algorithms make gene prediction from multiple features of gene-coding sequences. Methods on purely statistical grounds, using either probabilistic or pattern-based schemes to score candidate genes, display high sensitivity for discovering genes without a match. Most *ab initio* gene prediction programs distinguish coding (CDS) and non-coding sequences (NCDS) with their differences in nucleotide composition, intron splice sites, promoters, translational start/stop sites, and polyadenylation signals. These signals are generally integrated for evaluating the coding likelihood of a sequence. However, SPs prediction tools could not take all the characteristics into consideration. The integration of multiple criteria decreases the chance that false exons are predicted as true (low false-positive rate) but likely increases the chance that true exons are not predicted (high false-negative rate). The issue of false-negative prediction is particularly serious for smaller CDSs (≤300 nucleotides) due to the difficulty in distinguishing the relatively few biologically meaningful sequences from the very large pool of small ORFs (sORFs) (Basrai et al., 1997). Noting the relatively high false-negative rate of current gene finding algorithms and the difficulty to identify SP genes, recent studies focus only one or two signals to predict sORFs and they also take considerations from functional constraints in the follow-up analysis. Hanada et al. developed the program package sORF finder for identifying sORFs according to the nucleotide composition bias among coding sequences and the potential functional constraint at the AA level through evaluation of KA/KS ratio, because a functional coding sORF is expected to undergo stronger selective constraints on non-synonymous sites than for synonymous ones. Yang XH et al. identified candidate sORFs set by protein domain-scanning that is searching the InterPro database for annotated protein domain/motifs (Yang et al., 2011). The codon adaptation index (CAI), which is based on the similarity of usage of preferred and a limited number of codons for highly expressed genes, has been used to evaluate the coding potential of a putative ORF (Sharp and Li, 1987). Hanada et al. used this simple gene-finding method in a large-scale search for sORFs encoding proteins of 30–100 AA in the intergenic regions of the Arabidopsis genome (Hanada et al., 2007). On the basis of this research, Hanada et al. developed the program package sORF finder for identifying sORFs according to the nucleotide composition bias among coding sequences and the potential functional constraint at the AA level through evaluation of synonymous and non-synonymous substitution rates (Hanada et al., 2010). This measurement becomes less robust for sORFs, because the parameter will fluctuate dramatically as ORF length decreases. Only 2% of un-annotated sORFs predicted by Hanada et al. (2007) were confirmed by the Arabidopsis proteomic data (Castellana et al., 2008). Later, Termier and Kalogeropoulos examined the probability of functionality of sORFs and described computational techniques based on a combination of codon usage, AA composition, and dipeptide frequencies in the encoded protein to distinguish coding and non-coding sequences (Termier and Kalogeropoulos, 1996). In addition, Xiaohan Yang et al. reported an integrative sORF discovery strategy based on transcriptomics, proteomics, and computational biology, which was validated by both bioinformatics (e.g., protein domain-scanning) and experimental approaches (e.g., protein mass spectrometry) (Yang et al., 2011). Although multiple criteria could minimize the risk of considering a fortuitous ORF to be a meaningful protein-coding gene, it is also hardly to achieve efficient designation of small coding sequences from the very large pool of sORFs because of the growing error of short sequence annotation.

### Evidence-based strategies

Many experimental strategies have been used as gene prediction or validation tools and these methods have the ability to predict novel genes that could not be identified *in silico*. Most of them are based on gene expression data, such as RNA-Seq, EST (Expressed Sequence Tags), DNA microarray, and SAGE (serial analysis of gene expression). Although expression cannot (at all) be used to validate the translation of a SP, it is still the effective approach to address a SP candidate. Recent studies show a squared Pearson correlation coefficient of ~0.40, which implies that ~40% of the variation in protein concentration can be explained by knowing mRNA abundances (Vogel and Marcotte, 2012). Yamada et al. have used *Arabidopsis thaliana* full-length cDNA data and EST data from *A. thaliana*, *Brassica*, rice, and wheat to pinpoint transcribed, un-annotated genomic regions to identify novel transcribed sequences in *A. thaliana* (Yamada et al., 2003). Each plausible gene can be identified if it matches with EST or cDNA sequence. But some coding sORFs may be either expressed under specific conditions not covered or tend to have significantly lower expression levels than long high expressional genes leading to few evidence of sORFs to be found in transcriptome experiments. Another approach that has been developed is a microarray-based method, which is often used as a gene validation tool. The core principle behind microarrays is hybridization between two DNA strands. A single “chip” or array contains probes to determine transcript levels for every known gene in the genome of one or more organisms simultaneously. Shoemaker et al. used microarrays to refine and validate computational gene predictions for the human genome and define full-length transcripts on the basis of co-regulated expression of their exons (Shoemaker et al., 2001). It can provide more accurate results and represents a powerful tool for identifying transcripts. Nevertheless, this method requires explicitly designed chips and some small transcripts might not be systematically defined to allow the creation of the required chips. The serial analysis of gene expression (SAGE) technique can provide quantitative gene expression data without the prerequisite of a hybridization probe for each transcript. The general goal of SAGE technique is similar to DNA microarray and the difference between SAGE and microarrays is that SAGE sampling is based on lists of short sequence tags, not on hybridization of mRNA output to probes. The tag-based gene expression profiling can measure the expression levels of known or unknown sequences. It has been designed to catalog transcripts including a small number of unpredicted sORFs on a genome-wide level in yeast genome studies (Basrai et al., 1997; Velculescu et al., 1997; Basrai et al., 1999). Although it has the advantage of greater sensitivity to low levels of expression, the number of sORFs identified will be limited by the number of tags analyzed, the physiological state from which they are isolated, and the restriction enzyme used to define tags (Basrai et al., 1997). In addition to traditional transcriptional methods mentioned above, next-generation sequencing refreshes the methodology of transcriptomics that is, it directly sequences transcriptomes. By using deep sequencing technologies to sequence cDNA, RNA-Seq has been developed to transcriptome profiling quantitatively (Wang et al., 2009). The expression levels determined by RNA-Seq, which does not suffer from problems with background noise, are more accurate than traditional cross-hybridization methods. If the sequencing depth is sufficient, RNA-Seq would discover novel transcripts especially for SPs, some of which are hardly to be detected effectively in traditional expression-based methods. Despite of the individual advantages and limitations, all of the expression-based methods have several potential problems to identify SPs because sensitivity is contingent on the extent of the expression datasets, which might exclude genes with little evidence or expressed in uncovered specific conditions. For example, only low-level expressors of the SP of DAP-5 could be selected by the transfections with the original episomal-based vector or with the bicistronic vector, because overexpression of the DAP-5 was lethal to HeLa cells (Levy-Strumpf et al., 1997). Another SP is negative p53, whose expression inhibits DEK RNA interference-induced p53 transcriptional induction, as well as cell death, thus directly implicating p53 activation in the observed apoptotic phenotype (Wise-Draper et al., 2006). Therefore, those low-level expressed SPs are difficult to be verified by expression-based methods. These problems would not be encountered by analysis of a collection of transposon insertions, which can identify genes expressed at different times in the life cycle and determine the subcellular locations of the encoded gene products as well as the phenotype of the disrupted strains. No cDNA is required and the majority of new genes are either short or overlap a previously un-notated gene on the opposite strand. Smith et al. described a genetic footprinting method based on the endogenous yeast transposon Ty1 (Smith et al., 1996). This method could be useful for identifying sORFs if primers against interfeature regions (regions lying between known ORFs, tRNA genes, or other sequence “features”). In gene-finding studies in yeast (Ross-Macdonald et al., 1999; Kumar et al., 2002), candidate genes are identified by means of large-scale shuttle mutagenesis (Seifert et al., 1986) with a modified transposon as a simple gene trap. However, genes encoding proteins below a 100 AA cutoff might be under-represented in a mutational search because of the small target size for mutagenesis and the number of insertions analyzed. In addition to the expression-based method and mutagenesis, mass spectrometry has allowed for large-scale surveys of the proteome. Yang XH et al. identified highest-confidence candidate sORFs set by proteomics data using protein mass spectrometry. Proteomics has now advanced sufficiently to allow for the systematic quantification of proteins. But it also excludes large amounts of protein-coding sORFs because of the fast and dynamic nature of biological process.

### Integrated strategies

**Figure 2:**
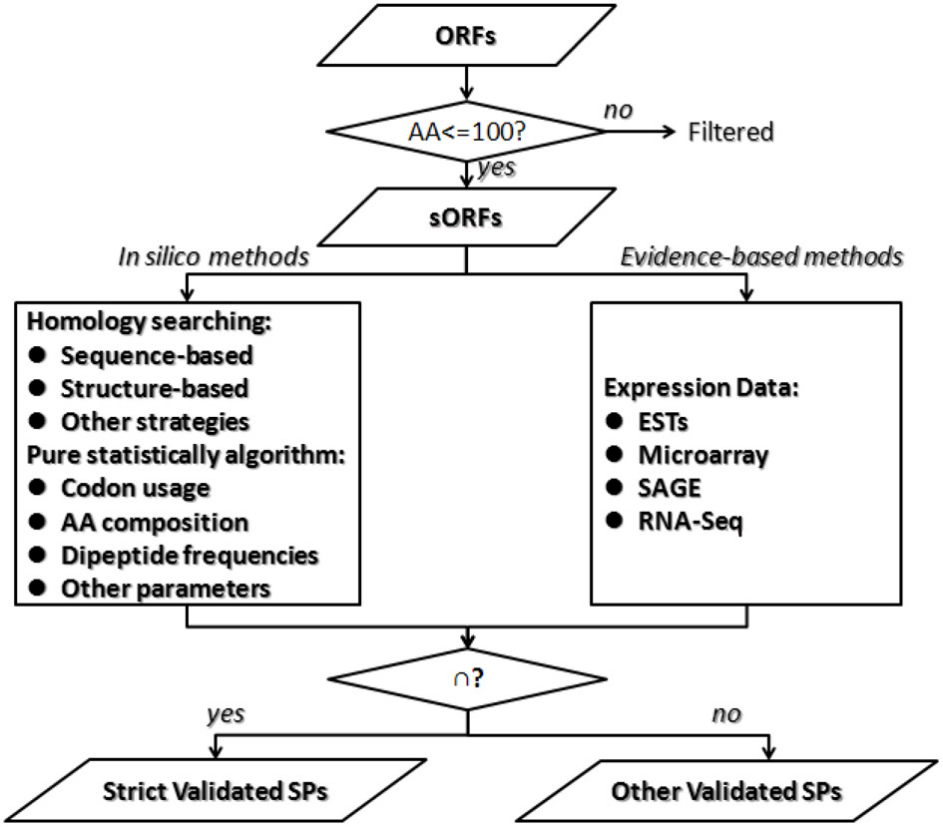
**An overview of integrated strategies for small proteins prediction**. It is challenge to differentiate meaningful gene-coding sORFs from inutile sORFs because the shorter the protein sequence, the greater the probability of error rate of detection. First we suggest splitting the annotation of SPs from other proteins. Second, it is better to combine both *in silico* algorithms and evidence-based analysis. Then merge the two parts of results and get two sets of SPs as follows. The strict validated SPs are those validated by both methods, while other validated SPs are those only validated by either *in silico* algorithms or evidence-based analysis.

As for the prediction of short protein-coding genes, the challenge is that short non-coding ORFs are difficult to distinguish from real genes; the shorter the protein, the greater the probability of error rate of detection. No single technique is comprehensive. In order to predict short genes completely and correctly, most studies combine both genome-wide searching algorithms *in silico* and expression analysis. Kumar et al. integrated methods of gene-trapping, microarray-based expression analysis, and genome-wide homology searching for finding overlooked sORFs and antisense sORFs in yeast (Ross-Macdonald et al., 1999; Kumar et al., 2002). The 137 genes discovered using this approach, including 104 SPs-coding genes, constitute 2% of the yeast genome and represent a wealth of overlooked biology. Yang et al. reported an integrative sORFs discovery strategy based on experimental data (transcriptome), coding potential prediction, evolutionary conservation, and gene family clustering (Yang et al., 2011). The sORF candidates predicted in this study display a relatively high rate of proteomics support and protein mass spectrometry support. We provided an overview of the integrated strategies for SPs prediction (Figure 2). In the computational stage, *in silico* programs could discover rarely expressed sORFs or tightly regulated sORFs, which are hardly detected in experimental methods. Homology searching methods are valuable for conserved SPs discovery but are not available for novel SPs candidates and some SPs share similar structure. Pure statistically algorithms combine multiple parameters and generate feature or pattern of SPs, which could be conducted from training set of SPs-coding genes in relative organisms. It is efficient for the designation of non-conserved small coding genes excluded by alignment-based methods. But computational methods include many false positives, some of which could be validation in the experimental stage. Expression-based approaches directly assess the gene expression level, which supplement to validate the meaningfulness of predicted sORFs. Experimental methods could also predict some new sORFs missed in computational stage. This integrated strategy could improve the sensitivity and specificity of annotating SPs-coding genes in stringent genomes, but the choice of each method applied and each parameter set is contingent.

## Genome-wide prediction in species level

Because of the advances in experimental and computational approaches, it has emerged that most studies (Table 2) are focused on large-scale discovery of SPs in species rather than a small number of SP families in specific organisms. In prokaryotes, Peter performed systematic function analysis of potential proteins, including 345 small polypeptide ORFs (of 85 codons or less) in *Bacillus subtilis*, which is known to produce an abundance of small polypeptides (Zuber, 2001). In single-cell eukaryotes, e.g., *S. cerevisiae*, Marco used genome-wide comparative analysis and identified 117 novel small genes, 84 of which are transcribed (Kessler et al., 2003). Kastenmayer et al. used gene expression-based analyses and homology searching and brought the total number of un-annotated sORFs in *S. cerevisiae* to 299, 247 of which have been verified experimentally (Kastenmayer et al., 2006). Earlier studies in plants showed that relatively little is known about sORF genes except for a number of small secreted proteins in *A. thaliana* (Cock and McCormick, 2001; Butenko et al., 2003). Recent studies have revealed a large number of novel coding sORFs. Lease and Walker predicted 33,809 un-annotated *Arabidopsis* ORFs encoding SPs of 25–250 AA in length (Lease and Walker, 2006); Hanada identified 3241 coding sORFs with either evidence of transcription or purifying selection which likely to be novel coding gene (Hanada et al., 2007). In animals, Emmanuel and Vini identified nearly 600,000 sORFs in the putatively non-coding euchromatic DNA of *Drosophila melanogaster* (Ladoukakis et al., 2011); Frith et al. reported that ~10% of proteins in *Mus musculus* are <100 AAs, although the majority of these are variants of proteins that are >100 AAs (Frith et al., 2006). Despite the inherent difficulties of identifying sORFs, these publications of large-scale discovery efforts may reveal additional sORFs with more valuable data and more advanced sORFs discovery methods.

**Table 2:** **Summary of large-scale sORF studies in different organisms**.

**Organism**		**Genome size (Mbp)**	**Protein-coding genes**	**sORFs^a^**	**Verified^b^**	**%^c^**	**Source**
Prokaryotes	*Bacillus subtilis*	4	4100	345	180	4	*Peptides*, 2001. 22(10)
Eukaryotes	*Saccharomyces cerevisiae*	12	5865	299	247	4	*Genome Res*, 2003. 13(2);
							*Genome Res*, 2006. 16(3)
	*Arabidopsis thaliana*	120	29,157	7159	3241	11	*Plant Physiol*, 2006. 142(3);
							*Genome Res*, 2007. 17(5)
	*Drosophila melanogaster*	180	13,907	4561	401	3	*Genome Biol*, 2011. 12(11)
	*Mus musculus*	2500	31,035	1240	1167	4	*PLoS Genet*, 2006. 2(4)

It describes studies focused on large-scale discovery of SPs in species and their results.

a Numbers of coding or annotated sORFs (<100 AA);

b Numbers of sORFs with experimental evidence or known function;

c The fraction of verified sORFs relative to previously annotated protein coding genes.

## Structural application for SPs

Except for functional importance, many studies have demonstrated that SPs containing <40 AAs with a compact, folded structure provide simple model systems for studying protein folding and stability as well as serving as scaffolds for the rational design of new functional motifs (Cunningham and Wells, 1997; DeGrado et al., 1999; Imperiali and Ottesen, 1999), which benefits both computational simulation and pharmaceutical studies.

In the simulation of protein folding, SPs are often used as model systems to overcome folding speed limits and to provide insight into the complex architecture of proteins. Generally, the polypeptide chains that are made up of thousands of atoms and hence consist of millions of possible interatomic interactions. It might be supposed that the resulting complexity would make the accurate prediction of protein structure and protein-folding mechanisms nearly impossible (Baker, 2000). However, SPs and domains can be folded quickly and correctly as the number of factors that influence folded state stability is reduced. As a result, many studies have used small motifs for structural simulation. Struthers et al. showed a metal-independent folded structure (ββα) reproduced in a 23 AA peptide through an iterative process (Struthers et al., 1996). Jennifer et al. designed a discretely folded SP motif based on the toxin hand (TH) motifs (Ottesen and Imperiali, 2001). Neidigh et al. have reported the smallest stable structural Trp-cage motif, a 20 AA peptide that adopts a well-defined globular shape, which provides a new tool for elucidating protein conformational preferences (Gellman and Woolfson, 2002; Neidigh et al., 2002; Qiu et al., 2002). The SP motif rapidly and accurately provides an excellent model for secondary structure simulation and provides the foundation for understanding the structures of large proteins.

The engineering of novel functional SPs has the potential to become a fundamental step toward the conversion of a protein functional epitope or a flexible peptide lead into a classical pharmaceutical. Such SPs represent a potential intermediate step in the development of drugs targeted to a protein–protein interface (Cunningham and Wells, 1997). The design of bioactive small molecules for interaction at large protein–protein interfaces remains a challenge and many studies are focused on minimizing proteins into significantly smaller polypeptides via both rational design processes and selection from vast combinational libraries (Martin and Vita, 2000). To date, scientists have designed a few SPs whose stability or instability has enhanced our understanding of those rules. Both of the natural (e.g., α/β scorpion toxin fold, protease inhibitors, leucine zipper, and zinc finger) and artificial SPs (TASP) have been used as structural scaffolds in the engineering of novel binding activity (Martin and Vita, 2000). Some of them can be used directly in therapy or exhibit a high potential to serve as drugs. In all cases, they represent precious structural intermediates that are useful as identification frameworks for peptidomimetic design or lead directly to new small organic structures, representing novel drug candidates.

## Evolution characterization of SPs

Proteins evolve under a variety of constraints, for example, as specific functions, base or AA compositions (Knight et al., 2001) and sequence length (Lipman et al., 2002). Studies of the evolutionary characterization of SPs draw attention to the question of how evolutionary trends affect variation of protein length. Two obvious observations from the evolutionary characterization of SPs are as follows. First, SPs are likely to change whereas long proteins are likely to be conserved. Studies (Guigo et al., 2003; Wei et al., 2005; Windsor and Mitchell-Olds, 2006) indicate that computational gene prediction methods are not generally capable of identifying SPs, which display elevated Ka/Ks ratios in interspecific comparisons, suggesting that SPs are generally rapidly evolving sequences. Furthermore, Lipman et al. studied the relationship between length and conservation (Lipman et al., 2002) and Zhao et al. analyzed SPs across eight Eukaryotes (Zhao et al., 2012). It is found that SPs tend to be non-conventional proteins and appear to have lineage-specific or tissue-specific function. There appears to be a significant evolutionary trend favoring shorter rather than longer proteins, possibly because of the need to minimize the cost of protein translation and the cost of the relationships that are required to fold longer, particularly multi-domain, proteins (Hartl and Hayer-Hartl, 2002). Perhaps too many changes in longer proteins would increase the risk of undesirable side-effects; i.e., deleterious interactions with other cellular components. The evolutionarily stable core of archaeal genomes includes the great majority of genes coding for conserved proteins involved in genome replication and expression, but only a relatively small subset of metabolic functions (Makarova et al., 1999). By contrast, the majority of SPs involve metabolic processes, transcriptional regulation or cell communication rather than essential roles in organisms. It is possible that vital functional proteins are more conserved than regulatory proteins in order to decrease side-effects, whereas poorly conserved proteins appear to tend toward minimal domain size and retain lineage-specific functions. Second, SPs are ancient and the origin of the protein universe is highly likely to have arisen from SPs with simple hydrogen-bonded, secondary structural elements instead of the details of side-chains. There is a tendency toward greater protein length along with increasingly complex genomes. Many prokaryotes generally have shorter proteins, on average, than eukaryotes (Makarova et al., 1999). Among the eukaryotes, proteins of the microsporidium *Encephalitozoon cuniculi*, which has an extremely compact genome, are smaller than the corresponding proteins in organisms with larger genomes (Katinka et al., 2001). There is evidence that a very small set of secondary structural elements, compacted from non-homologous representative proteins in the Protein Data Bank (PDB) of 41–150 residues, is complete for single-domain protein structures (Zhang et al., 2006). Similarly, we found that a very small set of SPs in the NCBI genpept cover a large number of domains in Pfam-A families (Table S2). These results support that SPs contain the majority, if not all, of the core secondary structural element, which can be used as the starting template. As SPs evolve, some could be folded into compact multi-domain proteins, whereas others could prefer to remain as small as originally created; at the same time, some new proteins are created along with species differentiation.

Above all, these two observations suggest that SPs might have important roles in evolutionary trends and give an possible answer to why nature needs SPs, but some intriguing questions that remain unanswered are focused on what a unique evolutionary pattern of SPs is and how an SP could reveal additional surprises.

## Conclusions and perspectives

SPs generally consist of a simple domain and tend to be treated as trivial molecules in biological processes. Large proteins have become priority targets to be analyzed whereas study of SPs is an almost untapped virgin territory in biological research. Despite an increasing number of SPs to be identified and involved in various biological functions, the vast majority of SPs are annotated as hypothetical proteins or proteins with function unknown. This is partly due to the limitations and challenges in most current gene discovery techniques, which are not generally appropriate for SPs identification. SPs have largely escaped detection and are hard to be differentiated from large amounts of artifacts. The integrated strategies combing *in silico* algorithms and evidence-based analysis could be more capable to discover potential SPs to some extent, although these strategies require improvements and it is also required more specific algorithms and techniques in this aspect produced in the future. Recent detection success suggests it is possible for large-scale identification and systematic analysis of SPs or sORFs at the genome level instead of only a limited number of SP families. The more exciting thing is scientists are gradually paying more attention toward solving exciting questions, such as why does nature need SPs if there are functional characterizations or unique evolutionary patterns for small peptides? The same question might arise for micro RNAs or small RNAs. Besides, smaller motifs have length advantages in iterative modeling, synthesis and structural characterization, prompting interest in discovering efficient testing procedures for pharmaceutical design strategies or principles.

## Conflict of interest statement

The authors declare that the research was conducted in the absence of any commercial or financial relationships that could be construed as a potential conflict of interest.
